# Patient satisfaction and survival of maxillary overdentures supported by four or six splinted implants: a systematic review with meta-analysis

**DOI:** 10.1186/s12903-021-01572-6

**Published:** 2021-05-08

**Authors:** Fabrizio Di Francesco, Gennaro De Marco, Estefani B. Capcha, Alessandro Lanza, Corina M. Cristache, Rolando Vernal, Emilio A. Cafferata

**Affiliations:** 1 Multidisciplinary Department of Medical, Surgical and Oral Sciences, School of Dentistry, Campania University Luigi Vanvitelli, Via Luigi De Crecchio 7, 80138 Naples, Italy; 2 Academic Department of Clinical Stomatology, Section of Implant Dentistry, Universidad Peruana Cayetano Heredia, Lima, Peru; 3 Department of Dental Techniques, Faculty of Midwifery and Medical Assisting (FMAM), Carol Davila University of Medicine and Pharmacy, Bucharest, Romania; 4 Periodontal Biology Laboratory, Faculty of Dentistry, Universidad de Chile, Santiago, Chile; 5 Department of Periodontology, School of Dentistry, Universidad Científica del Sur, Av. Paseo De La República 5544, Miraflores, Lima, Peru

**Keywords:** Patient satisfaction, Overdenture, Splinted design, Dental implant, Systematic review

## Abstract

**Background:**

Implant-supported overdentures offer enhanced mechanical properties, which lead to better patient satisfaction and survival rates than conventional dentures. However, it is unclear whether these satisfaction levels and survival rates depend on the number of implants supporting the overdenture. Therefore, this systematic review aimed to compare maxillary overdentures supported by four or six splinted implants in terms of patient satisfaction, implant survival, overdenture survival, and prosthodontic complications.

**Methods:**

Cochrane Central Register of Controlled Trials (CENTRAL), MEDLINE (PubMed), and EMBASE databases were systematically searched and complemented by hand searching from 2000 to 2019, employing a combination of specific keywords. Studies comparing the use of four versus six implants for supporting overdentures with at least one-year of follow-up after prosthesis installation and including ten fully edentulous patients were included. The risk of bias (RoB) was analyzed with Cochrane’s RoB 2 and Newcastle–Ottawa tools. Implants and prosthesis survival rates were analyzed by random-effects meta-analysis and expressed as risk ratios or risk differences, respectively, and by the non-parametric unpaired Fisher’s test.

**Results:**

A total of 15 from 1865 articles were included, and reported follow-up times after implant placement ranged from 1 to 10 years. Irrespective of the number of implants used, high scores were reported by all studies investigating patient satisfaction. Meta-analysis and non-parametric Fisher’s test showed no statistical differences regarding the survival rate of implants (*P* = 0.34, *P* = 0.3) or overdentures (*P* = 0.74, *P* = 0.9) when using 4 versus 6 splinted implants to support overdentures, and no significant differences regarding prosthodontic complications were found between groups. Randomized studies presented high RoB and non-randomized studies presented acceptable quality.

**Conclusions:**

Within the limits of this systematic review, we can conclude that the bar-supported overdenture on four implants is not inferior to the overdenture supported by six implants for rehabilitating the edentulous maxilla, in terms of patient satisfaction, survival rates of implants and overdentures, and prosthodontic complications.

**Supplementary Information:**

The online version contains supplementary material available at 10.1186/s12903-021-01572-6.

## Background

Edentulism is recognized as a physical disability that severely compromises nutrition, speech, self-esteem, and perceived aesthetics. Conventionally, fully edentulous patients have been frequently rehabilitated with complete dentures; however, due to progressive maxillary bone loss, these patients often experience a lack of prosthetic retention, stability, and chewing difficulty, which negatively affect their oral health-related quality of life [1]. Instead, when alternative implant-supported overdentures are chosen, the functional shortcomings associated with the use of conventional dentures are mostly overcome, resulting in improved patient satisfaction, comfort, and masticatory performance [2]. Indeed, both maxillary and mandible implant-supported overdentures have been indicated as the prime treatment of choice for patients with persistent complaints regarding the retention and stability of their conventional dentures, and insufficient residual tissue support [2, 3].

Although the fear of pain, limited mobility, and treatment cost are important barriers considered for this treatment, the optimal cost–benefit ratio offered by the maxillary implant-supported overdenture (MIOD) prosthodontic rehabilitation, i.e. performing the least number of interventions and, consequently, using the minimum number of implants for the patient’s optimal oral rehabilitation, surpasses its limitations [3, 4]. Nevertheless, there are no current clinical guidelines clearly indicating an ideal number and position of implants, or the attachment systems for supporting maxillary overdentures, as opposed to the case of mandibular overdentures, in which a large body of evidence, for instance, recommends the colocation of at least two implants for supporting them [5–7].

In this context, different systematic reviews have suggested that MIODs should be supported by at least four implants [3–9]. Though, other studies also encourage the use of six implants supported MIODs when there is sufficient bone, in order to enhance prosthesis’ stability and survival [10, 11]. Apart from that, the use of splinted implants for MIOD design has also been suggested when non-parallelism among implants occurs, palateless overdentures are realized, short implants are employed, or the opposing arch consists of natural teeth or fixed implant-supported prosthesis [4, 5, 12, 13].

Since the use of both four or six splinted implants with a bar anchorage for supporting a MIOD has been recommended, the question of whether six splinted implants supporting a MIOD may produce better patient satisfaction and treatment outcomes is a topic that remains unresolved [4, 6, 14]. Therefore, the aim of this systematic review was to compare maxillary overdentures supported by four or six splinted implants in terms of patient satisfaction, implant survival, overdenture survival, and prosthodontic complications.

## Methods

The Preferred Reporting Items for Systematic Reviews and Meta-Analyses (PRISMA) statement was used as a guideline to perform and report this systematic review [15]. The PICO research question was: “In fully edentulous patients (P) requiring a maxillary implant-supported overdenture (I), is there a difference between using four splinted or six splinted implants (C) in terms of patient satisfaction, implant and overdenture survival, and prosthodontic complications (O)*?”.*

### Search strategy

The Cochrane’s Central Register of Controlled Trials (CENTRAL), MEDLINE (via PubMed), and EMBASE databases were used in order to perform an electronic search between January 2000 and December 2019. The search strategy used the combination of the following keywords: (4-implant-retained OR 4 implant-supported OR 6-implant-retained OR 6-implant-supported OR implant-supported OR implant-retained) AND (maxillary overdenture OR splinted overdenture OR overdenture). Moreover, the reference lists of the most recent related systematic reviews were screened for the identification of additional eligible studies.

### Data selection, extraction, and analysis

Two reviewers (F.D. and G.D.), independently and in duplicate, assessed the titles and abstracts to determine their initial potential inclusion.

The following inclusion and exclusion criteria were adopted for studies:

- At least ten fully edentulous patients, rehabilitated with MIOD supported by four or six splinted implants.
- At least one of the following clinical parameters, such as patient satisfaction scores, implants survival rate, overdentures survival rate, and prosthodontic complications, in relation to MIOD supported on four or six splinted implants, was reported.
- At least one-year of follow-up after prosthesis installation.
- Human randomized controlled trials (RCTs), prospective studies, and retrospective studies were considered acceptable.
- Animal and in vitro studies were excluded.
- Studies using non-splinted implants were excluded.

Language or publication status were not considered for exclusion.

Data extraction from the included studies and data checking, to assure data extraction accuracy, were realized by the first independent reviewer (F.D.) and by a third independent reviewer (CM.C.), respectively. In particular, data were divided according to the number of placed splinted implants per prosthesis for the analysis of implants and overdentures survival rates.

### Risk of bias and quality assessment of studies

The reviewers (F.D. and G.D.) independently and in duplicate assessed the quality of the included studies. The Cochrane’s Risk of Bias Version 2 (RoB 2) tool, which assesses the randomisation process, deviations from intended interventions, missing outcome data, measurement of the outcome and selection of the reported result, was employed to analyze the included RCTs [16]. The quality of nonrandomized clinical studies was assessed using the Newcastle–Ottawa Scale (NOS) [17]. This scale uses a star system, in which a study is judged on three broad perspectives: The selection of the study groups (up to 4 points), the comparability of the groups (up to 2 points), and exposure or outcome of interest for case–control or cohort studies respectively (up to 3 points). Studies that met five or more of the Newcastle–Ottawa Scale score criteria were considered as good quality. For other types of studies, the quality-assessment was evaluated through a tool focusing on eight items developed by den Hartog et al. [18]. The studies scoring five or more pluses were considered acceptable.

### Statistical analysis

Inter-examiner agreement was assessed by Cohen’s Kappa (κ). A coefficient κ > 0.5 was considered acceptable for both the selection and RoB phases of the review. Dental implants and overdentures survival rates were expressed risk ratios (RR) or risk differences (RD) with 95% confidence intervals (CI) for dichotomous data, and as mean percentages (M%) and standard errors (SE) for continuous data. Due to the methodological and visually evident heterogeneity between studies, survival rates of implants and overdentures were analyzed by random-effects meta-analysis using the Mantel–Haenszel method for dichotomous data, and by the non-parametric unpaired Fisher’s test for continuous data. The review manager (RevMan) software version 5.2 (The Cochrane Collaboration) was used to plot forest plots. Statistically significant differences were established at *P* < 0.05.

## Results

### Selection of studies

**Fig. 1:**
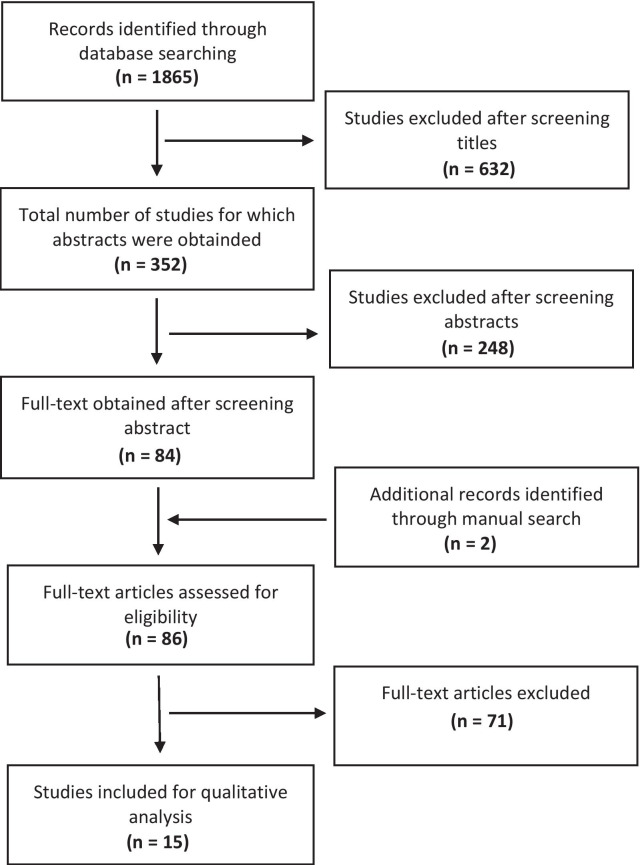
Data selection flowchart

The flowchart of data selection is shown in Fig. 1. A total of 1865 articles published were found from electronic searches. Two independent reviewers (F.D. and G.D.) carried out the screening and the selection process for the studies. All titles were checked, and 352 articles were selected for abstract reading. Then, the analysis of the abstracts excluded 268 articles that did not satisfy the eligibility criteria. Thus, 84 full-text articles were identified. In addition, checking the reference lists of the most recent systematic reviews produced two full-text studies, resulting in a total of 86 articles. Finally, 15 full-text articles satisfied the inclusion criteria, resulting in eight prospective studies, one retrospective study, and six RCTs. The main reasons for exclusions were: Not assessing implant-supported overdentures, not comparing 4 vs 6 implants and different outcome measures. Reviewers (F.D. and G.D.) achieved a κ = 0.8 inter-examiner agreement during selection.

**Fig. 2:**
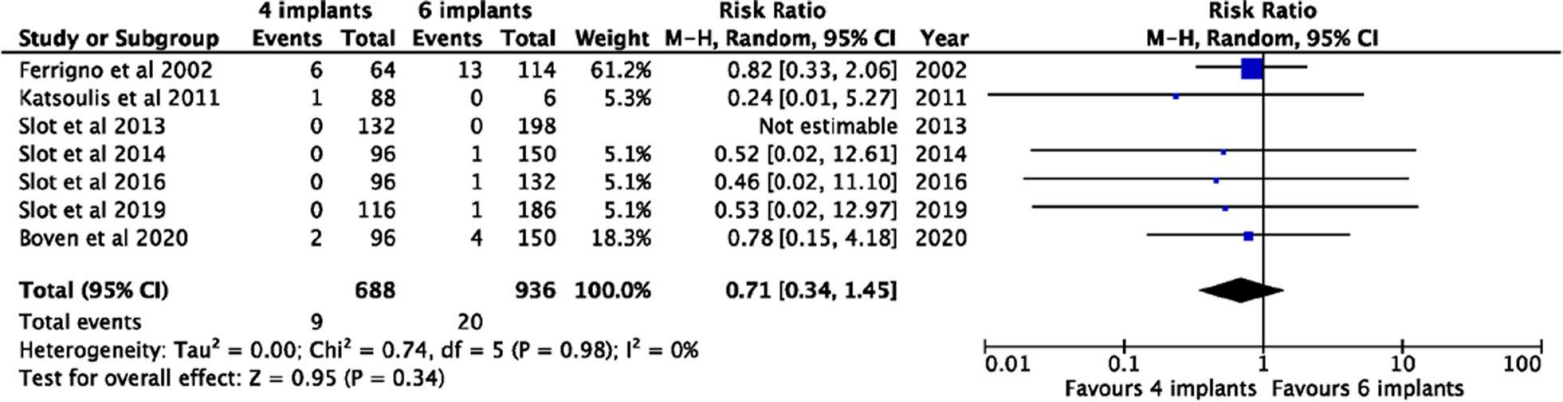
Forest plot of the dental implants survival risk ratios when using 4 versus 6 splinted implants for supporting overdentures

**Fig. 3:**
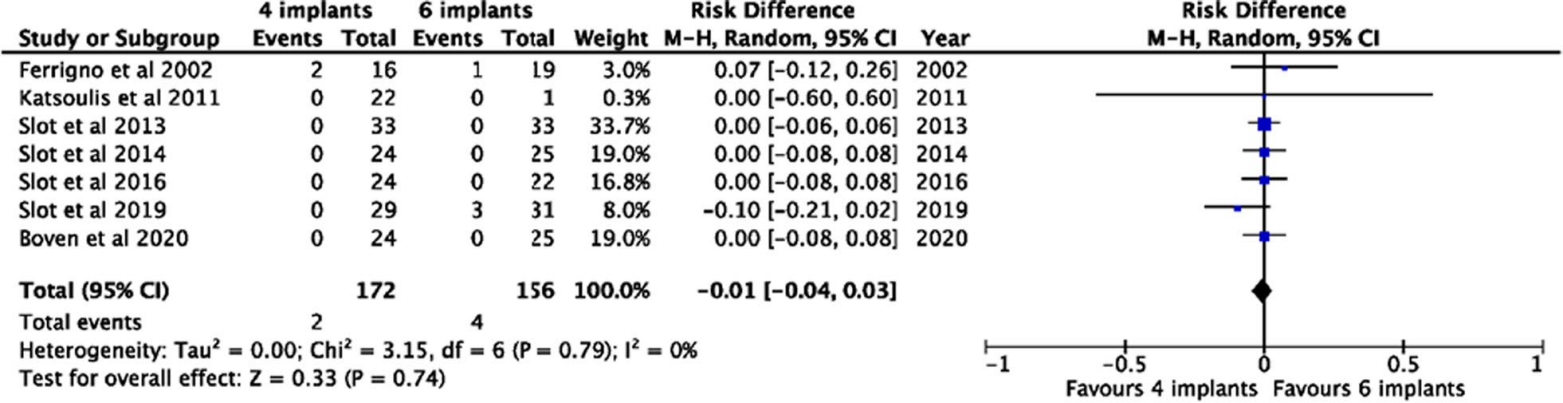
Forest plot of the overdentures survival risk diferences when using 4 versus 6 splinted implants for supporting them

**Fig. 4:**
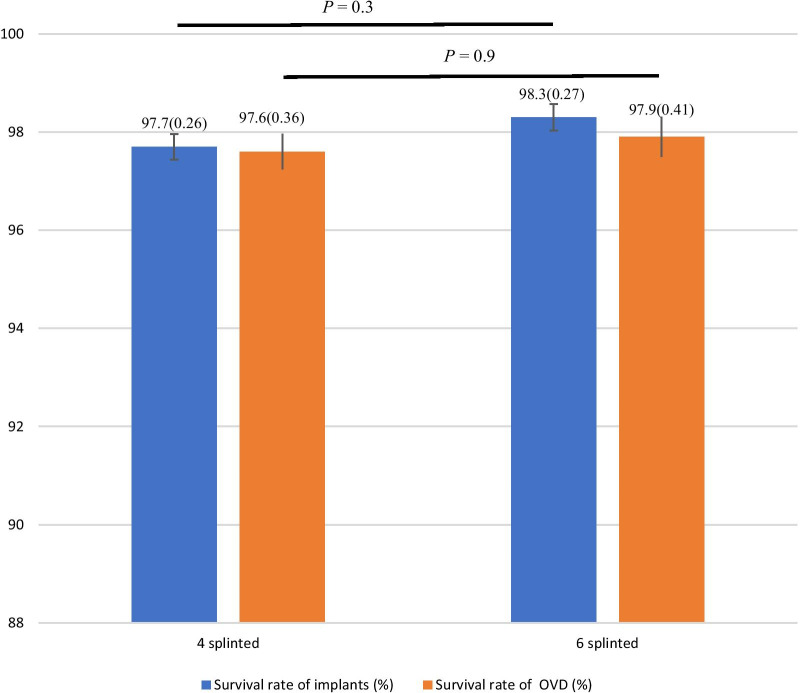
Dental implants and overdentures survival rates when using 4 or 6 splinted implants

Features of the included studies are reported in Table 1. Then, data were divided and analyzed into the group of 4 splinted implants (Table 2) and the group of 6 splinted implants (Table 3), respectively. Subsequently, data were statistically analyzed according to the number of implants placed, as reported in Figs. 2, 3 and 4. Only studies directly comparing the use of 4 versus 6 implants for supporting maxilla overdentures were included in the meta-analysis [10, 19–23, 28].

**Table 1:** Main characteristics extracted from the included studies

Study	Year	Study design	No. implants for patient, anchorage system	No. patients	OVD design	Opposing arch	System used for estimation of patient-reported results (Score range)
Boven et al. [23]	2020	RCT	4, bar	24	Palateless	Implant-retained overdenture	10-point rating scale (> 8)
Park et al. [31]	2019	RCT	4, bar	16	Full palatal coverage	ND	10-point rating scale (> 9)
Slot et al. [19]	2019	RCT	4, bar 6, bar	29 31	Palateless Palateless	Implant-retained overdenture Implant-retained overdenture	10-point rating scale (> 8) 10-point rating scale (> 8)
Slot et al. [20]	2016	RCT	4, bar 6, bar	24 22	Partial coverage Partial coverage	Implant-retained overdenture Implant-retained overdenture	10-point rating scale (> 8) 10-point rating scale (> 8)
Slot et al. [21]	2013	RCT	4, bar 6, bar	24 25	Palateless	Implant-retained overdenture	10-point rating scale (> 8) 10-point rating scale (> 8)
Slot et al. [22]	2014	RCT	4, bar 6, bar	33 33	Palateless Palateless	Implant-retained overdenture Implan-retained overdenture	10-point rating scale (> 8) 10-point rating scale (> 8)
Boven et al. [24]	2017	Prospective	6, bar ( anterior) 6, bar ( posterior)	25 25	Palateless Palateless	Natural teeth Natural teeth	10-point rating scale (> 8) 10-point rating scale (> 8)
Krennmair et al. [25]	2008	Retrospective	4, bar	16	Palateless	Implant-retained overdenture (ND) Fixed partial denture (ND) Natural teeth (ND)	Likert scale 1–5 (> 4.6)
Zou et al. [26]	2013	Prospective	4, bar	10	ND	ND	Likert scale 0–2 (1–2)
Mangano et al. [27]	2014	Prospective	4, bar	28	Palateless	Implant-retained overdenture	ND
Katsoulis et al. [28]	2011	Prospective	4, bar 6,bar	22 1	Palateless Palateless	Tooth-implant-supported- fixed prosthesis (ND) Natural teeth (ND)	ND
Mangano et al. [29]	2011	Prospective	4, bar	38	Palateless	Implant-retained overdenture	ND
Akca et al. [30]	2010	Prospective	4, bar	11	ND	Implant-supported overdenture (4) Implant-supported fixed prosthesis (1) Tooth-supported removable denture (1) Tooth-supported fixed prosthesis (4) Natural teeth (1)	ND
Ferrigno et al. [10]	2002	Prospective	4, bar 6, bar	16 19	ND ND	ND ND	ND ND
Van Assche [32]	2012	Prospective	6, bar	12	Palateless	ND	ND

OVD: overdenture. ND: not determined

**Table 2:** Analysis of survival rates of implants and overdentures in case of 4 splinted implants

Study	No.implants for patient, location	Pre-implant bone augmentation	Anchorage system	Bar Fabrication	Follow up(months)	Total no. impl	Total no. lost impl	Survival rate of implants (%)	Total no. OVD	Survival rate of OVD (%)
Slot et al. [19]	4, posterior region	Sinus floor	Milled titanium bar with mesial extensions and gold retentive clips	Abutment level	60	116	0	100	29	100
Slot et al. [20]	4, anterior region	No	Milled titanium eggshaped bar with distal extensions	Abutment level	60	96	0	100	24	100
Slot et al. [21]	4, posterior region	Sinus floor	Milled titanium bar with mesial extensions, and gold retentive clips	Abutment level	12	132	0	100	33	100
Slot et al. [22]	4, anterior region	No	Milled titanium eggshaped bar with distal extensions	Abutment level	12	96	0	100	24	100
Boven et al. [23]	4, anterior region	Some sinus floor	Milled titanium eggshaped bar with distal extensions	Abutment level	12	96	2	97.9	24	100
Krennmair et al. [25]	4, anterior region	No	Titanium or gold bar with distal extensions and retentive clips	Abutment level	42	64	0	100	16	100
Zou et al. [26]	4, ND	No	Dolder gold bar	Abutment level	36	40	0	100	10	100
Mangano et al. [27]	4, anterior region	No	Cobalt-chrome bar, without extensions and gold retentive clips	Abutment level	36	112	3	97,4	28	93,3
Katsoulis et al. [28]	4, ND	No	Titanium or dolder gold bar with distal extension	Implant level	24	88	1	98,9	22	100
Mangano et al. [29]	4, anterior region	No	Eggshaped dolder gold bar with or without distal extensions	Abutment level	60	152	4	97,4	38	100
Akca et al. [30]	4, ND	No	Eggshaped dolder gold bar with distal extensions	Implant level	59	44	1	97,7	11	88
Ferrigno et al. [10]	4, anterior and posterior regions	Some sinus floor	Dolder bar	ND	120	64	6	86,9	16	87,5
Park et al. [31]	4, anterior region	No	Hader bar and bar clips	Abutment level	12	64	1	96.3	16	100

OVD: overdenture. ND: not determined

**Table 3:** Analysis of survival rates of implants and overdentures in case of 6 splinted implants

Study	No.implants for patient, location	Pre-implant bone augmentation	Anchorage system	Bar fabrication	Follow up (months)	Total no. impl	Total no.lost impl	Survival rate of impl.(%)	Total No. OVD	Survival rate of OVD (%)
Slot et al. [19]	6, posterior region	Sinus floor	Milled titanium bar with mesial extensions and gold retentive clips	Abutment level	60	186	1	99.5	31	90.9
Slot et al. [20]	6, anterior region	No	Milled titanium eggshaped bar with distal extensions	Abutment level	60	132	1	99.2	22	100
Slot et al. [21]	6, posterior region	Sinus floor	Milled titanium bar with mesial extensions and gold retentive clips	Abutment level	12	198	0	100	33	100
Slot et al. [22]	6, anterior region	No	Milled titanium eggshaped bar with distal extensions	Abutment level	12	150	1	99.3	25	100
Boven et al. [23]	6, anterior region	No	Milled titanium bar	Abutment level	60	150	4	97	25	ND
Boven et al. [24]	6, posterior region	Sinus floor	Milled titanium bar	Implant level	60	150	1	99.3	25	ND
Katsoulis et al. [28]	6, ND	No	Titanium bar with distal extensions	Implant level	24	6	0	100	1	100
Ferrigno et al. [10]	6, anterior and posterior regions	Some sinus floor	Milled bar	ND	120	114	3	92.2	19	94.7
Van Assche et al.^32^	6, anterior and posterior regions	No	Dolder bar	Abutment level	24	72	1	98.6	12	100

OVD: overdenture. ND: not determined

### Patient satisfaction

Eight of the included studies examined patient satisfaction, and all of them showed high scores [19–26]. Most of the studies used the Vervoorn et al. questionnaire for denture satisfaction [19–24], which uses a scale of complaints, and frequently in combination with a “chewing ability” [19–22] or OHIP-49 [23, 24] questionnaire, thus assessing mostly patients’ perceived prosthesis comfort while wearing it or masticating with it. All the prospective studies performed the satisfaction assessment before and after the overdenture installation, with additional controls varying from 6 to 12 months after delivery. In four RCTs, Slot et al. reported general satisfaction scores higher than 8-points (on a 10-point rating scale) at both 1- and 5-year of follow-up for MIODs supported on both four and six splinted implants [19–22]. Similarly, in two RCTs, Boven et al. reported an overall satisfaction score greater than 8-points (on a 10-point rating scale) for MIODs supported by four [23] and six splinted implants [24] at 1- and 5-year follow-up, respectively. Krennmair et al. [25] and Zou et al. [26], for patients rehabilitated with MIODs supported on four splinted implants, found scores higher than 4.5 (Likert scale with score 1–5) and higher than 1 (Likert scale with score 0–2), respectively. According to the results of the analyzed studies, satisfaction among rehabilitated patients is uniformly high, irrespective of the use of four or six splinted implants to support maxillary overdentures.

### Survival of implants

Implants initially placed that were still present at follow-up were included and analyzed. The outcomes of included studies showed 18 lost implants on a total of 1164 implants in 291 patients rehabilitated with MIODs on four splinted implants [10, 19–22, 24–31] and 12 lost implants on a total of 1158 implants in 193 patients rehabilitated with MIODs on six splinted implants [10, 19–22, 24, 28, 33]. The pooled risk ratio for implant survival (RR = 0.71; CI = [0.34, 1.45]) showed no statistical differences between using 4 versus 6 splinted implants for supporting maxilla overdentures (*P* = 0.34) (Fig. 2). Similarly, the survival rate of implants appeared to be very similar, 4 implants (M% = 97.7; SE = 0.26) and 6 implants (M% = 98.3; SE = 0.26), also showing no statistically significant differences between the two groups (*P* = 0.3), as shown in Fig. 4.

### Survival of overdentures

Overdentures initially placed that were still present at follow-up were included and analyzed. Most of the included studies reported a survival rate of overdentures of 100% for both studies using 4 splinted implants [19–22, 24–26, 28, 29, 31] and 6 splinted implants [10, 20–22, 28, 32]. Only five included studies reported a survival rate of overdentures lower than 95%, three studies using 4 splinted implants [10, 21, 29] and two studies using 6 splinted implants [10, 19]. However, the pooled risk differences for overdenture survival (RD =  − 0.01; CI = [− 0.04, 0.03]) showed no statistical differences between using 4 versus 6 splinted implants for supporting maxilla overdentures (*P* = 0.74) (Fig. 3). Similarly, no statistical differences were detected regarding the survival rate of overdentures supported by 4 implants (M% = 97.6; SE = 0.36) or 6 implants (M% = 97.9; SE = 0.41) (*P* = 0.9), as reported in Fig. 4.

### Prosthodontic complications

Several included studies analyzing MIODs on four splinted implants reported that the most frequent complication involved clip loosening or fracture, or changing the bar clips due to retention loss [23, 27, 29, 31]. Slot et al. [20], comparing MIODs supported by four or six splinted implants placed in the anterior region, showed that prosthetic complications during 5 years of follow-up revealed a small number of events, mostly being repair of the denture base or teeth. No new bars or new overdentures had to be made, and no significant differences were found between the two groups. However, Slot et al. [19], comparing MIODs supported by four or six splinted implants placed in the posterior region at 5-year follow-up, showed that three new overdentures were re-made in the six‐implant group due to excessive wear of the denture base and teeth, reporting 90.9% survival rate of the overdentures. Van Assche et al. [32], analyzing MIODs on six splinted implants at 2-year of follow-up, reported only screw untightening in two of twelve treated patients.

### Risk of bias and quality assessment

Outcomes of the RoB assessment of included studies are reported in Tables 4, and 5. All six RCTs studies revealed a high RoB in the randomization procress and some concerns regarding the deviation from the intended interventions, in particular when considering the general abcense of random-sequence generation and blinding of participants and personnel (Table 4). Nevertheless, interventions differentiated entirely by the number of placed implants could be hard, if not impossible to blind, including patient, personnel, or outcome assessment blinding; thus, these results should be interpreted with caution. According to the authors’ definitions [16], the overall ranking showed no studies with a low risk of bias. The analysis of the NOS reported scores ranging 6 to 8, as shown in Table 5, whereas the quality-assessment tool by den Hartog et al. [18] highlighted that all four analyzed articles had a score of 6 or more (Additional file 1: Supplemental Table 1). Reviewers (F.D. and G.D.) had a κ = 0.8 inter-examiner agreement.

**Table 4:** Quality of included randomized controlled trials (RCTs) using Cochrane’s RoB 2 tool

Study	Randomisation process	Deviations from intended invervention	Missing outcome data	Measurement of the outcome	Incomplete outcome data addressed	Selection of the reported result	Overall RoB
Boven et al. [23]	High	Low	Some concerns	Low	Low	Low	High
Park et al. [31]	High	Low	Some concerns	Low	Low	Low	High
Slot et al. [19]	High	Some concerns	Low	Low	Low	Low	High
Slot et al. [20]	High	Some concerns	Low	Low	Low	Low	High
Slot et al. [21]	High	Some concerns	High	Low	Low	Low	High
Slot et al. [22]	High	Some concerns	High	Low	Low	Low	High

Deviations from intended interventions (involving blinding) should be interpreted with caution

**Table 5:** Quality of included studies using the Newcastle–Ottawa Scale (NOS) tool

Study	Selection****	Comparability**	Outcome***	Score
Boven et al. [24]	****	*	***	8
Krennmair et al. [25]	****	*	**	7
Zou et al. [26]	****	*	***	8
Katsoulis et al. [28]	****	*	***	8
Ferrigno et al. [10]	****	**	**	8

Studies that met five or more of the NOS score criteria were considered as good quality

## Discussion

Among the systematically revised literature, the data seems unequivocal when four or six splinted implant-supported prostheses are analyzed referring to patient satisfaction, in which high scores are reported by either of the groups. The data analysis of the included studies indicated that patients appear to be equally satisfied with MIODs supported by four or six splinted implants. In addition, most of the included studies (11/15) reported a horseshoe design of overdentures in both groups [19–25, 27–29, 32]. Patients usually require an overdenture without palatal coverage in order to increase comfort, taste, phonation, pharyngeal control, salivary flow, and hygiene. Another issue of discussion was the number of dental implants recommended to be installed to support a maxillary overdenture [33]. In the literature, it seems that the minimum favorable number to support a MIOD without palate coverage is four or six splinted implants [4–6, 33–36]. This concept is in line with several included studies in this review, reporting a survival rate of implants greater than or equal to 97%, both for palateless MIOD on 4 splinted implants [19–23, 25, 27–29] and palateless MIOD on 6 splinted implants [20, 21, 25, 26, 28, 29, 32]. Slot et al. highlighted that the implant-supported split bar anchorage system has a stronger influence on patient satisfaction than conventional dentures, supporting the splinted design over four or six implants [20, 21, 28, 29]. The reason lies in the concept that the splinted design offers more retention and stability and allows to realize a palateless MIOD, ensuring better predictability of treatment in terms of implant and overdenture survivals and patient satisfaction.

Several systematic reviews have proposed that implants supporting maxillary overdentures should be splinted in order to provide better force distribution on the prostheses, more retention, and stability when subjected to both vertical and oblique forces, and to avoid potential overloading of single implants [4, 5, 9, 31, 37]. In addition, implant-supported overdentures have been able to provide edentulous patients a stable centric occlusion and improved chewing capabilities [26, 34], irrespective of the number of implants placed and the opposing natural or artificial dentition [23, 24]. However, the question arises as to whether the number of splinted implants, 4 or 6, or their location is more important [20, 21, 28]. According to the data analysis of this systematic review, the analyzed studies investigating 4 splinted implants, employing both the anterior region [19, 21–23, 27, 28] and the posterior region, including the sinus [29], reported survival rates of implants higher than 97% and 96%, respectively. Similarly, the analyzed studies investigating 6 splinted implants employing both the anterior region [20, 21, 26] and the posterior region, including the sinus [19, 22, 26], reported survival rates of implants higher than or equal to 97% and 99%, respectively. Thus, no statistical difference was detected in the survival rate of implants between two analyzed groups irrespective of the implant installation zone. However, when sufficient bone in the anterior region is available, extensive bone augmentation procedures such as maxillary sinus floor elevation surgery could be prevented, meaning less treatment time, less morbidity, and few treatment costs [5, 13]. In addition, oral hygiene is easier to perform in the anterior region than in the posterior region, and the repaired bone defect after the often more extended augmentation procedures in the posterior region is less stable than in the anterior region [5, 13]. Therefore, whether the placement of 4 implants is chosen, the tendency is to place implants in the regions between the canine and second premolar, avoiding pneumatized maxillary sinuses and poor bone quality zones [5, 13].

As far as survival of the overdenture is concerned, data analysis of the included studies showed no significant differences between using 4 splinted implants or 6 splinted implants for supporting maxilla overdentures. However, apart from the survival of the prosthesis, which is always high and not sufficiently linked to the number of implants and the type of anchorage, it is important to analyze the success of the prosthesis influenced by the mechanical complications related to implant components (loosening or fracture of abutment or screw), and technical complications including issues related to anchorage structure (clip loosening or fracture, or bar fracture or lost) or prostheses (repairs of fractured prostheses or overdenture teeth) [38–42]. Indeed, in the work by Kiener et al., the increased tightening of the inner abutment screws was the most recurrent mechanical complication in bar supported maxillary overdentures [43]. Differences in maintenance reported between milled gold alloy bars and solid titanium bars could be attributed to the physical properties of the materials used [44]. Katsoulis et al. showed fractures of bars or extensions occurred more often with gold bars than titanium bars [28]. This is supported by the findings of Widbom et al. [45], who recommended a harder and more resistant metal alloy for superstructure construction than the gold alloy. Moreover, the design of the prosthesis should provide the optimal passive fit and stress distribution, especially in type III and type IV bone. Thus, from a mechanical point of view, the absence of abutments and the direct screw fixation of the bars at the implant-level could appear to be advantageous [23, 28, 30]. However, most of the included studies investigated bar design with abutment-level, reporting survivals of implants and overdentures greater than 96% for both 4 splinted implants [10, 19–23, 25–31] and 6 splinted implants [10, 19–23, 28, 32]. Nevertheless, the performance of more randomized clinical trials with low RoB, analyzing prosthodontic complications and comparing MIODs on 4 or 6 splinted implants connected at abutment-level or implant-level, is encouraged.

The clinical evidence found in this systematic review allows us to suggest that the choice between 4 or 6 splinted implants supporting a maxillary overdenture does not seem to be directly related to the clinical parameters detected. In light of these considerations, there was an indicative advantage in the use of 4 implants instead of 6 implants in order to reduce treatment costs, morbidity, and augmentation procedures. However, poor bone quality and quantity, reduced implant length and diameter, and consequently, low primary stability could lead to implant loss in the maxillae. In this context, if an implant is lost, the use of a 6 implant approach could avoid a new surgical intervention and would just need an adaptation of the overdenture. Contrarily, when an implant is lost in the 4 implants approach, a new implant and prosthesis suprastructure are often needed before the overdenture can be adjusted [47] Apart from that, treatment decision-making also deals with the choice of providing an implant-supported overdenture or a full-fixed prosthesis. In this context, 4 implants have also demonstrated to be sufficient for the long-term success of implant-supported full fixed prostheses [48], and achieving high levels of patients’ satisfaction [49]. Nevertheless, fully edentulous patients often present substantial bone and soft tissue deficiencies, which lead to prognathism, deficient facial support, speech disruption, and general esthetic problems that compromise the ubication of smile line and the length of the upper lip; thus, preventing the use of a fixed implant-supported prosthesis [50].

Therefore, the question: “Whether 6 splinted implants supporting a MIOD compared to 4 splinted implants may produce better treatment outcomes?” Still requires further investigation [13, 14, 19, 20]. This study was limited by the lack of prospective randomized clinical trials with a low RoB comparing maxillary overdentures supported by 4 or 6 splinted implants and considering the possibility to overcome previously reported blinding difficulties. In particular, there were five RCTs [10, 19–22] comparing 4 and 6 splinted group in this systematic review. However, of these five studies [10, 19–22], four derived from the same authors, and it appears that these studies represent only two studies with data published at 1 and 5 years each [19–22]. Thus, only three RCTs could be included in the quantitative analysis. Moreover, substantial heterogeneity between the studies and lack of data prevented the performance of quantitative assessment of patients’ satisfaction. In addition, this study is limited to only two treatment options from the universe of therapeutic modalities that comprehends implant-supported maxillary prostheses for fully edentulous patients, such as the use of 8- or more implants, zygomatic implants, and adjunct tissue augmentation procedures.

## Conclusion

Within the limits of this systematic review, it is concluded that the bar-supported overdenture on 4 implants is not inferior to the bar-supported overdenture on 6 implants in terms of patient satisfaction, implants or overdentures survival rates, and prosthodontic complications. However, future research, especially long-term analysis comparing maxillary overdentures supported by 4 or 6 splinted implants, is required in order to further clarify this issue.

## Supplementary Information

**Additional file 1: Supplemental Table 1.** Quality of included studies using the den Hartog et al. tool.

## Data Availability

Search results as well as data selection procedures for MEDLINE (Pubmed) (https://pubmed.ncbi.nlm.nih.gov), CENTRAL (https://www.cochranelibrary.com/central) and EMBASE (https://www.embase.com) databases are available on request.
