# Supplementary material for: Patient satisfaction and survival of maxillary overdentures supported by four or six splinted implants: a systematic review with meta-analysis
Source: BMC Oral Health. 2021 May 8;21:247. doi: 10.1186/s12903-021-01572-6 (PMC8106178; doi:10.1186/s12903-021-01572-6)
Supplement: Supplementary file 1 — Additional file 1: Supplemental Table 1. Quality of included studies using the den Hartog et al. tool. [file 12903_2021_1572_MOESM1_ESM.docx]

**Supplemental Table 1** Quality of included studies using the den Hartog et al tool.

| **Study** | **1.Are the characteristics of the study group clearly described?** | **2. Is there a high risk of selection bias? Are the inclusion and exclusion criteria clearly described?** | **3. Is the intervention clearly described? Are all patients treated according to the same intervention?** | **4.Are the outcomes clearly described? Are adequate methods used to assess the outcome?** | **5. Is blinding used to assess the outcome?** | **6. Is there a sufficient follow-up?** | **7. Can selective loss-to follow-up sufficiently be excluded?** | **8. Are the most important confounders or prognostic factors identified and are these taken into consideration with respect to the study design and analysis?** |
| --- | --- | --- | --- | --- | --- | --- | --- | --- |
| Mangano et al^27^ | + | + | + | + | + | + | + | - |
| Mangano et al^29^ | + | + | + | + | ? | + | + | - |
| Akca et al^30^ | + | + | + | + | - | + | + | + |
| Van Assche et al^33^ | + | + | + | + | + | + | + | - |

Studies scoring five or more pluses were considered acceptable.
